# 1986. Multicenter evaluation of *Staphylococcus aureus* prosthetic valve infective endocarditis with and without gentamicin treatment: Is it time to revisit?

**DOI:** 10.1093/ofid/ofac492.1611

**Published:** 2022-12-15

**Authors:** Tho H Pham, Kyle C Molina, Grace Erdman, Matthew A Miller, Vanthida Huang

**Affiliations:** Midwestern University College of Pharmacy-Glendale Campus, Glendale, Arizona; University of Colorado Hospital, Aurora, Colorado; University of Colorado Hospital, Aurora, Colorado; University of Colorado Hosppital, Aurora, Colorado; Midwestern University, College of Pharmacy-Glendale Campus, Glendale, Arizona

## Abstract

**Background:**

*Staphylococcus aureus* prosthetic valve infective endocarditis (SA-PVIE) is associated with high mortality. Gentamicin (GEN) with anti-staphylococcal antibiotics and rifampin are guideline recommendations for SA-PVIE extrapolated from *in vitro* data. GEN can lead to acute kidney injury (AKI), meanwhile, the clinical benefit on infection-related outcomes remains unclear. Therefore, we evaluated the impact of GEN on outcomes in SA-PVIE.

**Methods:**

This is a multicenter, retrospective cohort conducted at HonorHealth and UCHealth systems. Adults admitted between January 2014-2022 with definite/possible SA-PVIE by Duke Criteria were included if they received ≥2 days of treatment within 2 days of index culture. Cohorts were stratified by GEN receipt. The primary outcome was 90-day all-cause mortality. The secondary outcomes were treatment failure (change in antimicrobials, abscess development, new indication for cardiac surgery), 30-day all-cause mortality, and incidence of AKI by KDIGO Criteria.

**Results:**

Overall, 38 patients with definite (40%) and possible SA-PVIE (60%) met inclusion (13 GEN, 25 without GEN [no-GEN]). At baseline, 15 (40%) patients were in an ICU, median Pitt bacteremia score was 2, and methicillin-susceptible *S. aureus* predominated (71%). A total of 10 (26%) patients had valve surgery; median bacteremia duration was similar between GEN and no-GEN (4 vs 3 days, p = 0.26). Common antibiotics were vancomycin (95%), cefazolin (63%), and nafcillin (21%); rifampin was more common in GEN than no-GEN (20% vs 77%, p < 0.001). Baseline AKI (44% vs 46%) and renal impairment (8% vs 0%) were not different between GEN and no-GEN, respectively. GEN was initiated a median 3 days after index culture, most commonly as intermittent strategy (69%) with 3 mg/kg daily equivalent (84.6%). There was no statistical difference in treatment failure (23% vs 24%, p=0.17), 30-day mortality (20% vs 39%, p=0.22), or 90-day mortality (28% vs 43%, p=0.263) between GEN and no-GEN, respectively. Three in the (23%) GEN group experienced AKI, compared to 10 (40%) in no-GEN.

**Conclusion:**

We did not find that the addition of GEN to SA-PVIE therapy enhanced mortality benefit, yet patients without GEN may live to experience adverse events. Further studies were warranted.

**Disclosures:**

**All Authors**: No reported disclosures.

